# The lncRNA MALAT1 functions as a competing endogenous RNA to regulate MCL‐1 expression by sponging miR‐363‐3p in gallbladder cancer

**DOI:** 10.1111/jcmm.12920

**Published:** 2016-07-15

**Authors:** Shou‐Hua Wang, Wen‐Jie Zhang, Xiao‐Cai Wu, Ming‐Zhe Weng, Ming‐Di Zhang, Qiang Cai, Di Zhou, Jian‐Dong Wang, Zhi‐Wei Quan

**Affiliations:** ^1^ Department of General SurgeryXinhua HospitalShanghai Jiao Tong University School of MedicineShanghaiChina

**Keywords:** gallbladder cancer, MALAT1, competing endogenous RNA, miR‐363‐3p, MCL‐1

## Abstract

Gallbladder carcinoma (GBC) is an aggressive neoplasm, and the treatment options for advanced GBC are limited. Recently, long non‐coding RNAs (lncRNAs) have emerged as new gene regulators and prognostic markers in several cancers. In this study, we found that metastasis‐associated lung adenocarcinoma transcript 1 (MALAT1) expression was up‐regulated in GBC tissues (*P* < 0.05). Luciferase reporter assays and RNA pull down assays showed that MALAT1 is a target of miR‐363‐3p. Real‐time quantitative PCR and Western blot analysis indicated that MALAT1 regulated Myeloid cell leukaemia‐1 (MCL‐1) expression as a competing endogenous RNA (ceRNA) for miR‐363‐3p in GBC cells. Furthermore, MALAT1 silencing decreased GBC cell proliferation and the S phase cell population and induced apoptosis *in vitro. In vivo,* tumour volumes were significantly decreased in the MALAT1 silencing group compared with those in the control group. These data demonstrated that the MALAT1/miR‐363‐3p/MCL‐1 regulatory pathway controls the progression of GBC. Inhibition of MALAT1 expression may be to a novel therapeutic strategy for gallbladder cancer.

## Introduction

Gallbladder carcinoma (GBC) is the most common tumour of the biliary tract and is also the fifth most common gastrointestinal malignancy. Chemoresistance is the most notable characteristic of GBC, and the prognosis of patients with advanced GBC is dismal, with 5‐year survival rates of approximately 20% 1, 2. Therefore, it is urgent to investigate the molecular and biological functions underlying GBC progression, which may help to identify novel diagnostic and therapeutic targets.

Long non‐coding RNAs (lncRNAs) are a class of non‐coding RNAs longer than 200 nucleotides. Although the functions of most lncRNAs have not currently been characterized, recent reports have shown that they are involved in important biological functions and various pathological conditions, including cancer progression 3, 4.

Metastasis‐associated lung adenocarcinoma transcript 1 (MALAT1) is a widely expressed lncRNA that is greater than 8000 nucleotides in length. MALAT1 was first identified as a prognostic marker of patient survival in stage I non–small‐cell lung cancer 5. Recently, studies have suggested that MALAT1 is involved in cell cycle progression and tumorigenesis in various types of cancer, including hepatocellular carcinoma 6, gastric cancer 7, cervical cancer 8, clear cell kidney carcinoma 9 and oesophageal squamous cell carcinoma 10. Qi *et al*. found that Malat1 bound EZH2, inhibited the tumour suppressor PCDH10 and promoted gastric cell migration and invasion 11. Hu *et al*. verified that MALAT1 promoted colorectal cancer cell proliferation, invasion and metastasis *in vitro* and *in vivo* by increasing the expression of A‐kinase anchor proteins 9(AKAP‐9) 12. To date, only one article has reported that MALAT1 might serve as an oncogenic lncRNA that promotes proliferation and metastasis of GBC 13. Therefore, the roles of MALAT1 in GBC progression need to be further explored.

In this study, MALAT1 expression was shown to be up‐regulated in gallbladder cancer tissues, and knockdown of MALAT1 inhibited cell proliferation, reduced the proportion of cells in the S phase and induced cell apoptosis. Moreover, using luciferase reporter assays, we further confirmed that MALAT1 functions as a competing endogenous RNA to regulate Myeloid cell leukaemia‐1 (MCL‐1) expression by sponging miR‐363‐3p. The MALAT1/miR‐363‐3p/MCL‐1 regulatory network may be a novel therapeutic target for gallbladder cancer.

## Materials and methods

### Patients and samples

Thirty‐three GBC tissue samples and matched adjacent normal gallbladder tissue samples were obtained from patients with GBC who had undergone surgery between January 2010 and December 2011 in Eastern Hepatobiliary Surgical Hospital (Second Military Medical University, Shanghai, China) and Xinhua Hospital (Shanghai Jiao Tong University School of Medicine, Shanghai, China). All cases were reviewed by a pathologist and histologically confirmed as gallbladder cancer. Gallbladder carcinoma patients were staged according to the tumour node metastasis staging system (the 7th edition) of the American Joint Committee on Cancer. Patients recruited in this study received no other treatments prior to surgery. All samples were snap frozen in liquid nitrogen and stored at −80°C prior to RNA isolation. Informed consent was obtained from all patients. The data do not contain any information that could identify the patients. This study was approved by the Human Ethics Committee of Xinhua Hospital at Shanghai Jiao Tong University (Shanghai, China).

### Cell culture

The human gallbladder cancer cell lines SGC‐996 and NOZ were purchased from the Health Science Research Resources Bank (Osaka, Japan) and the cell bank of the Chinese Academy of Science (Shanghai, China), respectively. The non‐tumorigenic human intrahepatic biliary epithelial cell line H69 was purchased from the Health Prescience Resources Bank. Cells were cultured in DMEM (Gibco BRL, Grand Island, NY, USA) supplemented with 10% foetal bovine serum (HyClone; Invitrogen, Camarillo, CA, USA), 100 μg/ml penicillin and 100 μg/ml streptomycin (Invitrogen, Carlsbad, CA, USA). Cells were incubated at 37°C with 5% CO_2_.

### RNA preparation, reverse transcription and qPCR

Total RNA was prepared from gallbladder cancer cells and cancer tissues using TRIzol (TaKaRa, Dalian, China). Random primers and oligo (dT) were used in the reverse transcription reactions according to the manufacturer's protocol (TaKaRa). The reactions were incubated at 95°C for 60 sec., followed by 40 cycles of 95°C for 5 sec. and 60°C for 34 sec. Real‐time PCR was performed using a SYBR Green PCR kit (TaKaRa), and real‐time RT‐PCR reactions were performed on an ABI 7500 system (Applied Biosystems, Carlsbad, CA, USA). GAPDH and U6 were used as internal controls for lncRNAs and microRNAs, respectively. The primer sequences used were as follows: GAPDH (forward), 5′‐GTCAACGGATTTGGTCTGTATT‐3′ and GAPDH (reverse), 5′‐AGTCTTCTGGGTGGCAGTGAT‐3′; MALAT1 (forward), 5′‐ATGCGAGTTGTTCTCCGTCT‐3′ and MALAT1 (reverse), 5′‐TATCTGCGGTTTCCTCAAGC‐3′; MCL‐1 (forward), 5′‐GCTTGCTTGTTACACACACAGGTC‐3′ and MCL‐1 (reverse), 5′‐GCAGAACAATCAGCAATTTCAAGG‐3′; MiR‐363‐3p (forward), 5′‐GGCGGAATTGCACGGTATCC‐3′. The relative expression fold change of mRNAs was calculated by the 2^−ΔΔCt^ method. All experiments were performed in triplicate.

### Cell proliferation assays

The Cell Counting Kit‐8 (CCK‐8) assay was performed according to the manufacturer's protocols with SGC‐996 and NOZ cells (Beyotime, Shanghai, China), briefly, after transfection of si‐NC, si‐MALAT1, miR‐363‐3p inhibitor and si‐MALAT1 + miR‐363‐3p inhibitor, respectively. A total of 4000 cells in 100 μl complete medium were seeded into 96‐well plates (in triplicate for each group). After incubation for 24, 48, 72 and 96 hrs, 10 μl CCK‐8 assay solution was added to each well. Then, after incubation for another 2 hrs, optical density at 450 nm was measured with an enzyme immunoassay analyser (Thermo Fisher Scientific, Inc., Waltham, MA, USA) to estimate cell proliferation among different groups.

### Flow cytometric analysis

Flow cytometric analysis was performed to analyse the cell cycle. NOZ cells (1.5 × 10^5^) transfected with si‐NC, si‐MALAT‐1, a miR‐363‐3p inhibitor or si‐MALAT1 + miR‐363‐3p inhibitor were plated in six‐well plates. After a 48‐hr incubation, the cultures were incubated with propidium iodide for 30 min. in the dark. Cultures were collected, and the cell cycle was analysed using a flow cytometer (FACSCalibur; BD Biosciences, San Jose, CA, USA) after propidium iodide staining. Data were evaluated as a percentage distribution of cells in G0/G1, S and G2/M phases. The cultures were stained using annexin V–fluorescein isothiocyanate (Beyotime, Haining, China), and apoptosis rates were analysed using a flow cytometer (FACSCalibur; BD Biosciences, Sparks, MD, USA). The experiment was independently repeated three times.

### Western blot analysis

Whole cell extracts were prepared by lysis of cells in Radio‐Immunoprecipitation Assay (RIPA) buffer [1 mM ethylenediaminetetraacetic acid (EDTA), 150 mM NaCl, 50 mM 4‐(2‐hydroxyethyl)‐1‐piperazineethanesulfonic acid (HEPES), pH 7.4] with freshly added 0.01% protease inhibitor cocktail (Sigma‐Aldrich, Shanghai, China) followed by incubation on ice for 20 min. The supernatant (55 μg of protein) was separated with 12% SDS‐PAGE and electrophoretically transferred to a polyvinylidene fluoride membrane (Millipore, Shanghai, China), followed by incubation with antibodies against MCL‐1 (1:1000; Proteintech, USA) and GAPDH (1:1000; (Proteintech, Chicago IL,USA)). Blots were incubated with anti‐rabbit secondary antibody (1:3000; Beyotime, Shanghai) and visualized using enhanced chemiluminescence (Thermo Scientific, Shanghai, China). All experiments were performed in triplicate.

### RNAi and transfection

Two MALAT1‐siRNAs were purchased from Genepharm (Shanghai, China). A negative control siRNA was also provided by Genepharm. The siRNA sequences are as follows: si‐MALAT1‐1, sense‐1, 5′‐CACAGGGAAAGCGAGTGGTTGGTAA‐3′; antisense‐1, 5′‐TTACCAACCACTCGCTTTCCCTGTG‐3′; si‐MALAT1‐2, sense‐2, 5′‐GAGGUGUAAAGGGAUUUAUTT‐3′; antisense‐2, 5′‐AUAAAUCCCUUUACACCUCTT‐3′; negative control, sense, 5′‐UUCUCCGAACGUGUCACGUTT‐3′; antisense, 5′‐ACGUGACACGUUCGGAGAATT‐3′. The concentrations of relative siRNAs, mimics and inhibitors were 20 μM, and the working concentration was 20 nM. siRNA plasmids were transfected into cells using Lipofectamine^™^ 2000 (Invitrogen, Carlsbad) and were incubated for 48 hrs. The working concentration of the relative plasmids was 100 nM. The miR‐363‐3p mimic and inhibitor were transfected into cells using Lipofectamine^™^ 2000 (Invitrogen, Carlsbad). The Lv‐sh‐MALAT1 sequences were as follows: sense, 5′‐CACAGGGAAA GCGAGTGGTT GGTAA‐3′; antisense, 5′‐TTACCAACCACTCGCTTTCC CTGTG‐3′. The sequence of the negative control shRNA was 5′‐TTCTCCGAAC GTGTCACGT‐3′. The shRNAs were synthesized and inserted into the pHBLV‐U6 lentivirus core vector containing a ZS green fluorescent protein (Hanbio, Shanghai, China).

### Luciferase reporter assay

SGC‐996 and NOZ cells (2.0 × 10^4^) grown in a 96‐well plate were co‐transfected with 150 ng of empty pmir‐GLO‐NC, pmir‐GLO‐MALAT1‐Wt or pmir‐GLO‐MALAT1‐Mut (Sangon Biotech,Shanghai,China) and 2 ng of pRL‐TK (Promega, Madison, WI, USA) with a miR‐363‐3p mimic or miR‐NC into cells using Lipofectamine 2000 (Invitrogen,Carlsbad, California, USA). The relative luciferase activity was normalized to *Renilla* luciferase activity 48 hrs after transfection. The transfection was independently repeated three times.

### RNA‐binding protein immunoprecipitation assay

RNA immunoprecipitation (RIP) assays were performed using an EZ‐Magna RIP^™^ RNA‐Binding Protein Immunoprecipitation Kit (Milli‐pore,Billerica, MA, USA) according to the manufacturer's instructions. NOZ cells at 80–90% confluence were lysed in complete RIP lysis buffer, and 100 μl of whole cell extract was then incubated with RIP buffer containing magnetic beads conjugated to human anti‐Ago2 antibody (Proteintech, China). The negative control was normal mouse IgG (Beyotime, Shanghai,China), and the positive control was SNRNP70 (Millipore, USA). The co‐precipitated RNAs were isolated by TRIzol reagent (TaKaRa) and were detected by reverse transcription PCR. Total RNAs (input controls) and IgG were assayed simultaneously to demonstrate that the detected signals were the result of RNAs specifically binding to Ago2.

### Biotin‐labelled miRNA pull‐down assays

RNA pull‐down assays were performed as described previously 14. NOZ cells were transfected with biotinylated miR‐363‐3p, biotinylated miR‐363‐3p‐mut and biotinylated NC. Cells were collected at 48 hrs. The cells lysates were incubated with M‐280 streptavidin magnetic beads (Invitrogen, San Diego, CA, USA). To prevent non‐specific binding of RNA and protein complexes, the beads were coated with RNase‐free bovine serum albumin and yeast tRNA (both from Sigma‐Aldrich). The beads were incubated at 4°C for 3 hrs and washed three times with ice‐cold lysis buffer and once with high salt buffer (0.1% SDS, 1% Triton X‐100, 2 mM EDTA, 20 mM Tris‐HCl, pH 8.0 and 500 mM NaCl). The bound RNAs were purified using TRIzol for the analysis.

### Xenograft mouse model

NOZ cells (1.5 × 10^6^) stably expressing control shRNA or shRNA‐MALAT1 were subcutaneously injected into either side of the flank area of 4‐week‐old male athymic nude mice (*n* = 5 mice per group). Mouse tumour volumes were measured (0.5 × length × width^2^) weekly. The nude mice were killed, and the tumour tissues were excised and fixed in 4% paraformaldehyde solution. Tumour tissues were snap frozen in liquid nitrogen and stored at −80°C prior to RNA isolation for further study after 4 weeks. All animal experiments were performed in the animal laboratory centre of Xinhua Hospital and in accordance with the *Guide for the Care and Use of Laboratory Animals* published by the US National Institutes of Health (NIH publication number 85‐23, revised 1996). The protocol was approved by the Animal Care and Use Committee of Xinhua Hospital.

### Statistical analysis

All data are expressed as the mean ± S.D. from at least three separate experiments. The gene expression level of lncRNA‐MALAT1 in tumours was compared with adjacent normal tissues using a Wilcoxon test. The differences between groups were analysed using Student's *t*‐test. The difference was statistically significant at *P* < 0.05.

## Results

### MALAT1 is overexpressed in gallbladder cancer tissues

**Figure 1:**
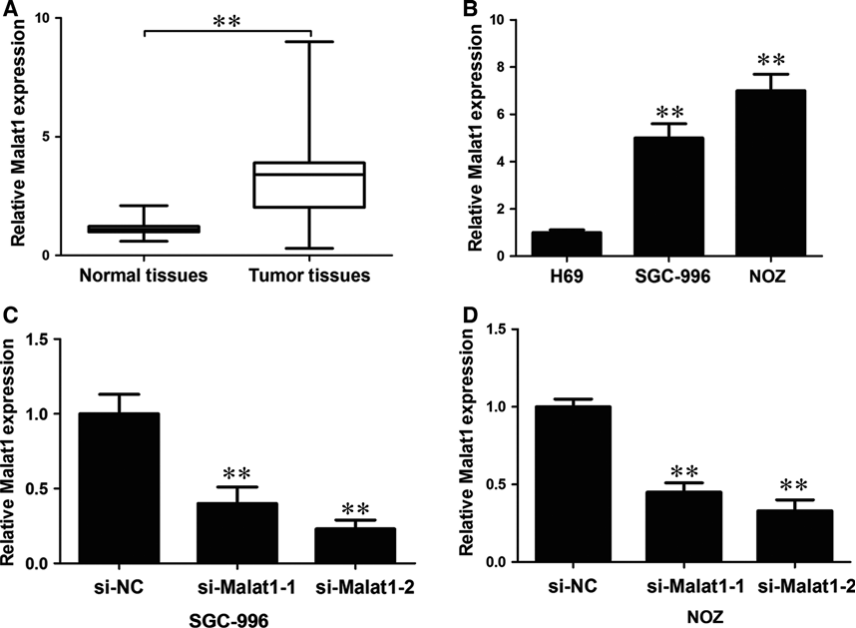
MALAT1 is overexpressed in gallbladder cancer tissues. (**A**) MALAT1 expression levels in GBC and adjacent normal tissues were detected by qRT‐PCR (*n* = 33). The relative expression fold change of mRNAs was calculated by the 2^−ΔΔCt^ method. The expression of MALAT1 was normalized to GADPH. The statistical differences between samples were analysed with paired samples *t*‐test. (**B**) MALAT1 expression levels in the GBC cell lines SGC‐996 and NOZ were detected by qRT‐PCR and compared with those in the human gallbladder epithelium cell line H69. The expression of MALAT1 was normalized to that in H69. (**C** and **D**) SGC‐996 and NOZ cells were transfected with two different siRNAs against MALAT1. Error bars represent the mean ± S.D. of triplicate experiments, **P < 0.05.

To explore the potential role of MALAT1 in human gallbladder cancer, we analysed 33 pairs of human gallbladder cancer tissues and matched adjacent non‐cancerous tissues. qRT‐PCR analysis revealed that MALAT1 expression levels were significantly increased in gallbladder cancer tissue samples compared to those of adjacent normal tissues (Fig. 1A). To evaluate the possible role of MALAT1 in GBC, we compared MALAT1 expression in two gallbladder cancer cell lines (SGC‐996 and NOZ cells) and a human gallbladder epithelium cell line H69 (Fig. 1B) and found that expression was significantly increased in the cancer lines. We transfected gallbladder cancer cell lines with two different siRNAs against MALAT1. Both siRNAs could efficiently knockdown the endogenous MALAT1 (Fig. 1C and D). The siRNA‐MALAT1‐2 was used in the later experiment to efficiently silence MALAT1.

### MiR‐363‐3p is a target of MALAT1

**Figure 2:**
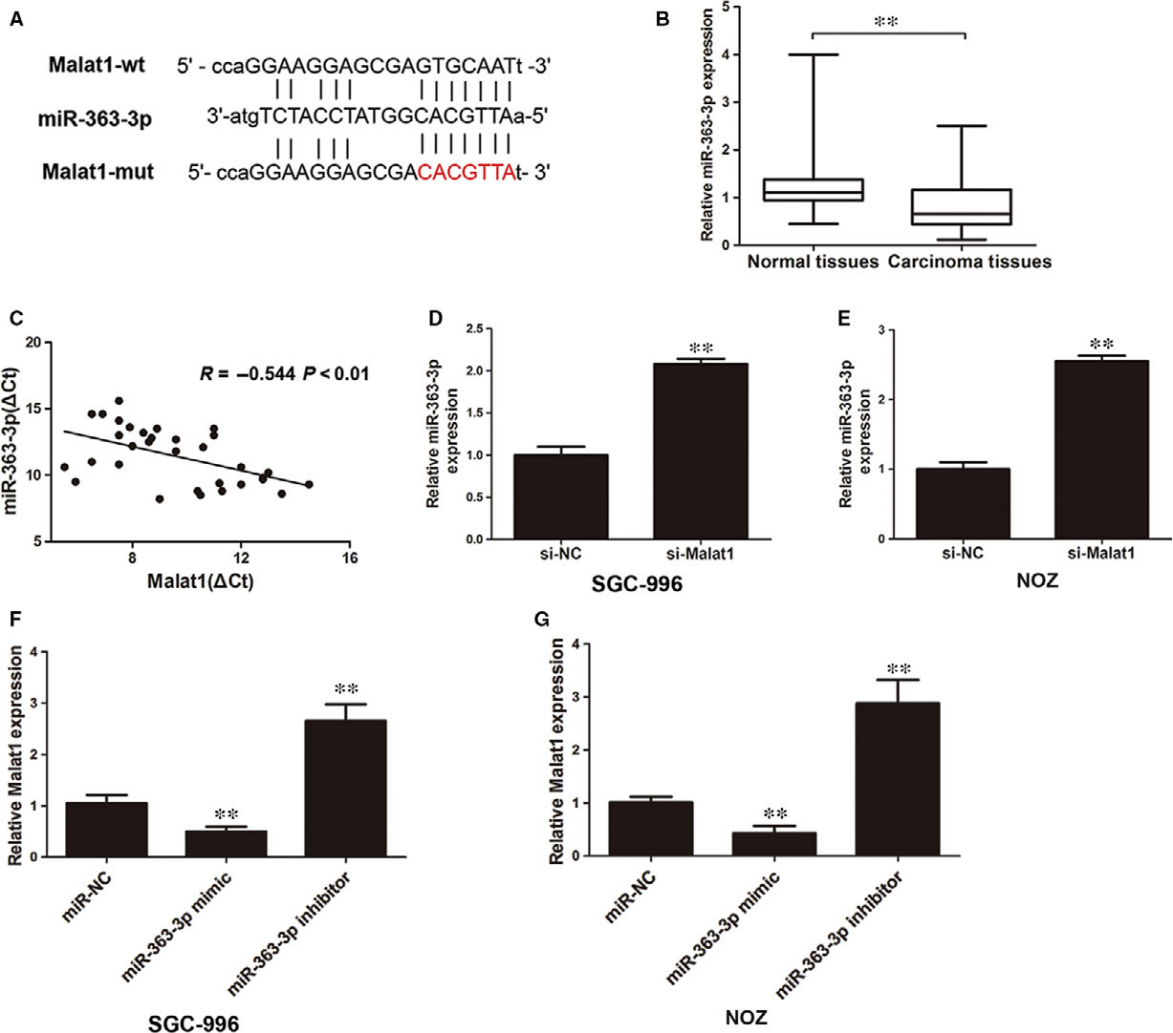
Identification of miR‐363‐3p as a target of MALAT1. (**A**) Alignment of potential MALAT1 sequences with miR‐363‐3p as identified by Starbase v2.0 (http://starbase.sysu.edu.cn/mirLncRNA.php). MALAT1 mutated at the putative binding site. (**B**) MiR‐363‐3p is down‐regulated in gallbladder cancer tissues compared to levels in adjacent normal tissues as determined by qRT‐PCR. The expression of miR‐363‐3p was normalized to U6. (**C**) The correlation between MALAT1 expression level and miR‐363‐3p level was measured in 33 gallbladder cancer tissues (*R* = −0.544, *P* < 0.01). The ΔCt values were subjected to Pearson's correlation analysis. (**D** and **E**) The expression of miR‐363‐3p was up‐regulated after silencing MALAT1 in SGC‐996 and NOZ cells. (**F** and **G**) The expression of MALAT1 was decreased after transfecting the miR‐363‐3p mimic and was increased after transfecting the miR‐363‐3p inhibitor into SGC‐996 or NOZ cells. Error bars represent the mean +S.D. of triplicate experiments, **P < 0.05.

A previous study reported that MALAT1 may be an oncogenic lncRNA that promotes proliferation and metastasis of GBC 13. We explored the potential mechanisms underlying growth suppression after MALAT1 knockdown. Emerging evidence has confirmed that lncRNAs are competing endogenous RNAs (ceRNAs) or molecular sponges that modulate miRNAs 15. We performed a bioinformatics analysis using Starbase 2.0 (http://starbase.sysu.edu.cn) and found that miR‐363‐3p contains a binding site for MALAT1. The predicted binding sites for MALAT1 in the miR‐363‐3p sequence are illustrated in Figure 2A. qRT‐PCR results showed that miR‐363‐3p was down‐regulated in the tumour specimens (Fig. 2B), but MALAT1 was overexpressed in the same tumour tissues (Fig. 1A), suggesting a significant negative correlation between MALAT1 and miR‐363‐3p (*R* = −0.544, *P* < 0.01, Fig. 2C). We then examined the impact of MALAT1 silencing on miR‐363‐3p in both cell lines. MiR‐363‐3p was significantly increased by knockdown of MALAT1 (Fig. 2D and E). Next, we demonstrated that the expression level of MALAT1 was decreased after transfection with a miR‐363‐3p mimic and increased after transfection with a miR‐363‐3p inhibitor in SGC‐996 and NOZ cells (Fig. 2F and G).

**Figure 3:**
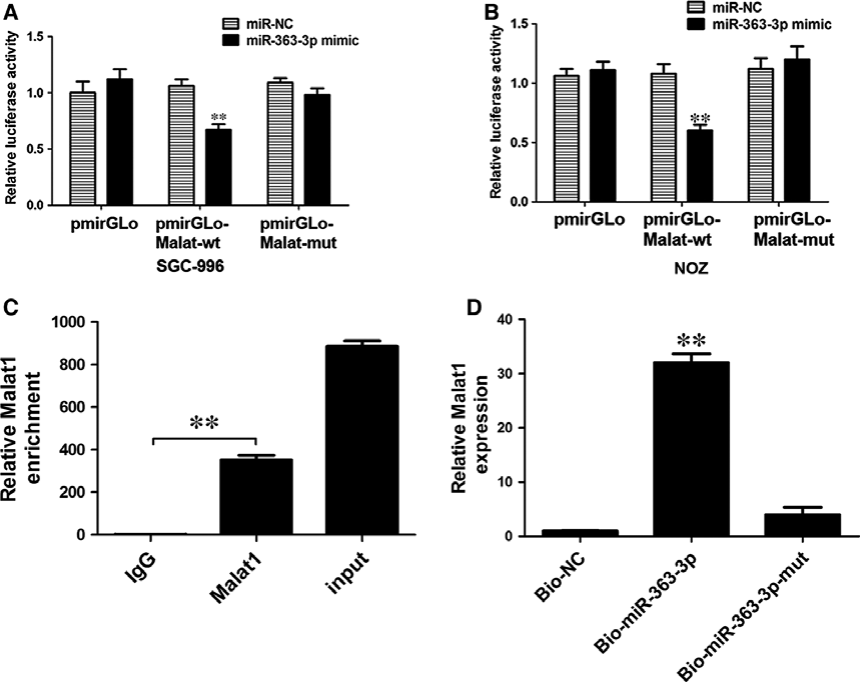
The underlying mechanism of the regulation of miR‐363‐3p and MALAT1. (**A** and **B**) Luciferase reporter activity in SGC and NOZ cells was detected after co‐transfection with miR‐363‐3p mimic and luciferase reporters containing nothing, wild MALAT1 or mutant transcripts. Data are presented as the relative ratio of firefly luciferase activity to *Renilla* luciferase activity. Error bars represent the mean ± S.D. of triplicate experiments. **P < 0.05. (**C**) Amount of MALAT1 bound to Ago2 or IgG measured by qRT‐PCR after RIP in NOZ cells (**P < 0.05). (**D**) NOZ cells were transfected with biotinylated NC (NC‐Bio), biotinylated WT miR‐363‐3p (miR‐363‐3p‐Bio) or biotinylated mutant miR‐363‐3p (miR‐363‐3p‐Mut‐Bio), and biotin‐based pull‐down assays were conducted after 48 h of transfection. MALAT1 levels were analysed by qRT‐PCR (**P < 0.05).

To further confirm that MALAT1 was a functional target of miR‐363‐3p, a dual‐luciferase reporter assay was performed with SGC and NOZ cells. Our results showed that the luciferase activity was significantly decreased by the co‐transfection of miR‐363‐3p mimic + pmiR‐Glo‐MALAT1‐Wt compared with the co‐transfection of miR‐363‐3p mimic + miR‐NC or miR‐363‐3p mimic + pmiR‐Glo‐MALAT1‐Mut in SGC and NOZ cells (Fig. 2A, Fig. 3A and B). These results demonstrated that MALAT1 is a target of miR‐363‐3p. MiRNAs function through the RNA‐induced silencing complex (RISC), which contains many associated proteins, such as the Argonaute (Ago) protein family. This family of proteins binds to the mature miRNA and guides it to the mRNA and plays a central role in RNA silencing. We next examined whether MALAT1 and miR‐363‐3p were in the same RISC by RIP assays and demonstrated enrichment of MALAT1 in NOZ cells (Fig. 3C). In addition, we carried out RNA pull‐down assays to determine whether miR‐363‐3p could directly bind to MALAT1. NOZ cells were transfected with biotinylated miR‐363‐3p and then harvested for biotin‐based pull‐down assays. As shown by qRT‐PCR, MALAT1 was pulled down by biotin‐labelled miR‐363‐3p oligos but not the mutated oligos (binding sites were mutated to the complementary sequences) that disrupted base pairing between MALAT1 and miR‐363‐3p (Fig. 3D). These results indicated that miR‐363‐3p directly bound to MALAT1.

### Knockdown of MALAT1 inhibits MCL‐1, a target of miR‐363‐3p

**Figure 4:**
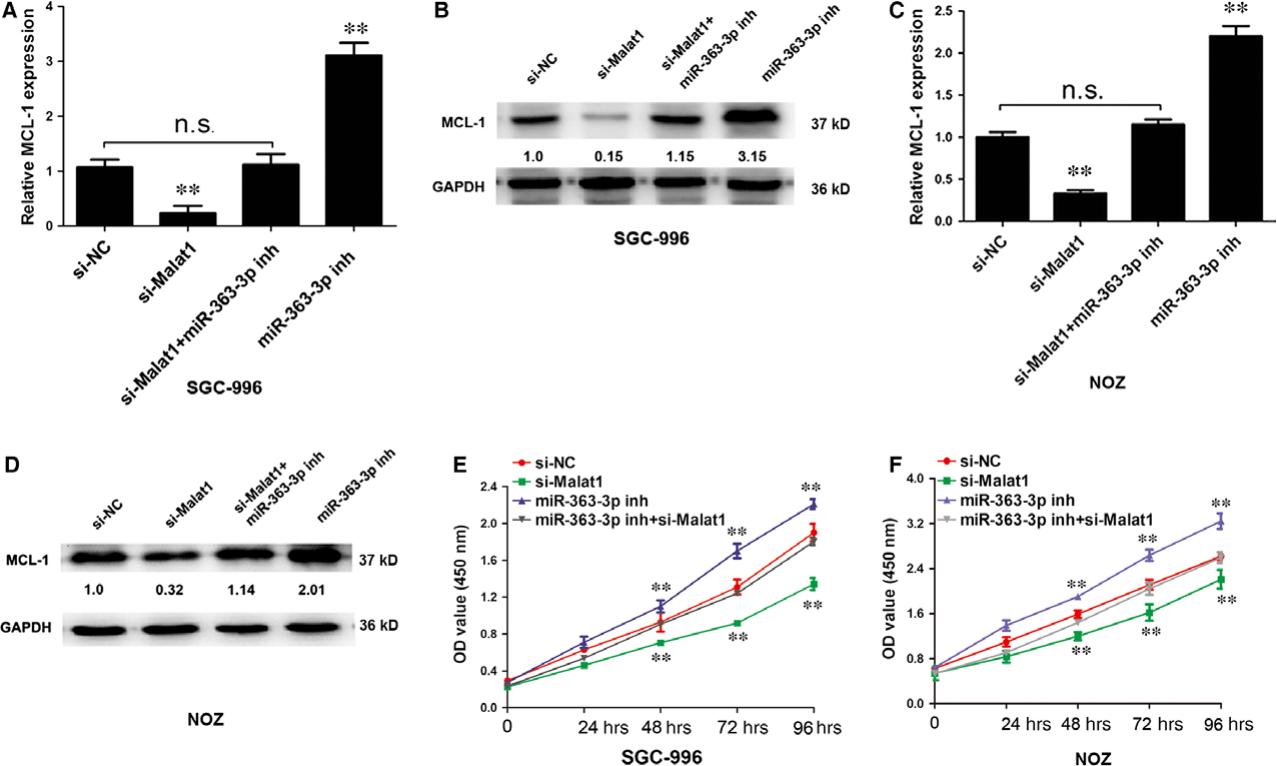
MCL‐1 is regulated by MALAT1. (**A** and **B**) The mRNA or protein levels of MCL‐1 in SGC‐996 cells transfected with si‐NC, si‐MALAT1, si‐MALAT1 + miR‐363‐3p inhibitor and miR363‐3p inhibitor. (**C** and **D**) The mRNA or protein levels of MCL‐1 in NOZ cells transfected with si‐NC, si‐MALAT1, si‐MALAT1 + miR‐363‐3p inhibitor and miR363‐3p inhibitor. (**E** and **F**) Cell proliferation was evaluated in SGC‐996 and NOZ cells transfected with si‐NC, si‐MALAT1, and miR‐363‐3p inhibitor and si‐MALAT1 + miR‐363‐3p inhibitor by CCK‐8 assays after cell transfection for 24, 48, 72 and 96 hrs. Absorbance at 450 nm is shown. Error bars represent the mean ± S.D. of triplicate experiments. **P < 0.05, n.s., not statistically significant.

Myeloid cell leukaemia‐1 was reported to be a target of miR‐363‐3p in a previous study 16. Both qRT‐PCR and Western blot analyses showed that knockdown of MALAT1 in SGC‐996 cells led to a significant decrease in endogenous MCL‐1 mRNA and protein expression. A miRNA‐363‐3p inhibitor reversed these effects, which indicated that MALAT1 partially modulates MCL‐1 by competing with miRNA‐363‐3p (Fig. 4A and B). Similar these results were found in NOZ cells (Fig. 4C and D). Together, these data indicated that by binding miR‐363‐3p, MALAT1 acts as a ceRNA‐targeting MCL‐1, modulating the MCL‐1 expression and imposing an additional level of post‐transcriptional regulation.

### MALAT1 promotes gallbladder cancer cell proliferation and inhibits cell apoptosis by competing with miRNA‐363‐3p *in vitro*

**Figure 5:**
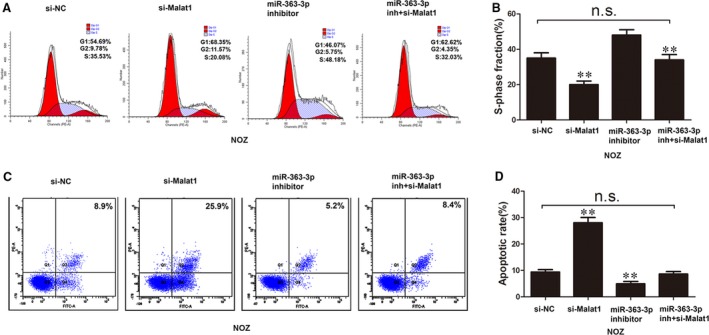
Cell cycle and apoptosis analyses in NOZ cells. (**A** and **B**) The cell cycle was evaluated in NOZ cells transfected with si‐NC, si‐MALAT1, and miR‐363‐3p inhibitor and si‐MALAT1 + miR‐363‐3p inhibitor. (**C** and **D**) Cell apoptosis was evaluated in NOZ cells transfected with si‐NC, si‐MALAT1, and miR‐363‐3p inhibitor and si‐MALAT1 + miR‐363‐3p inhibitor. Error bars represent the mean ± S.D. of triplicate experiments. **P < 0.05, n.s., not statistically significant.

To explore the role of MALAT1 in GBC cell progression, siRNA‐MALAT1 was stably introduced into SGC‐996 and NOZ cells. The growth curves detected by CCK‐8 assays showed that MALAT1 knockdown significantly decreased GBC cell proliferation, but this was reversed by co‐transfection of si‐MALAT1 and a miR‐363‐3p inhibitor in SGC‐996 and NOZ cells (Fig. 4E and F). Cell cycle assays confirmed that the number of cells in S phase was significantly reduced in the MALAT1 silencing group compared with the control group, but this was reversed by co‐transfection of si‐MALAT1 and the miR‐363‐3p inhibitor in NOZ cells (Fig. 5A and B). Annexin V‐FITC analysis showed that the proportion of the cell population undergoing apoptosis was increased after knockdown of MALAT1 in NOZ cells, but this was reversed by co‐transfection of si‐MALAT1 and the miR‐363‐3p inhibitor in NOZ cells (Fig. 5C and D).

### Knockdown of MCL‐1 inhibits gallbladder cancer cell proliferation, decreases the S phase cell population and induces cell apoptosis in NOZ cells

**Figure 6:**
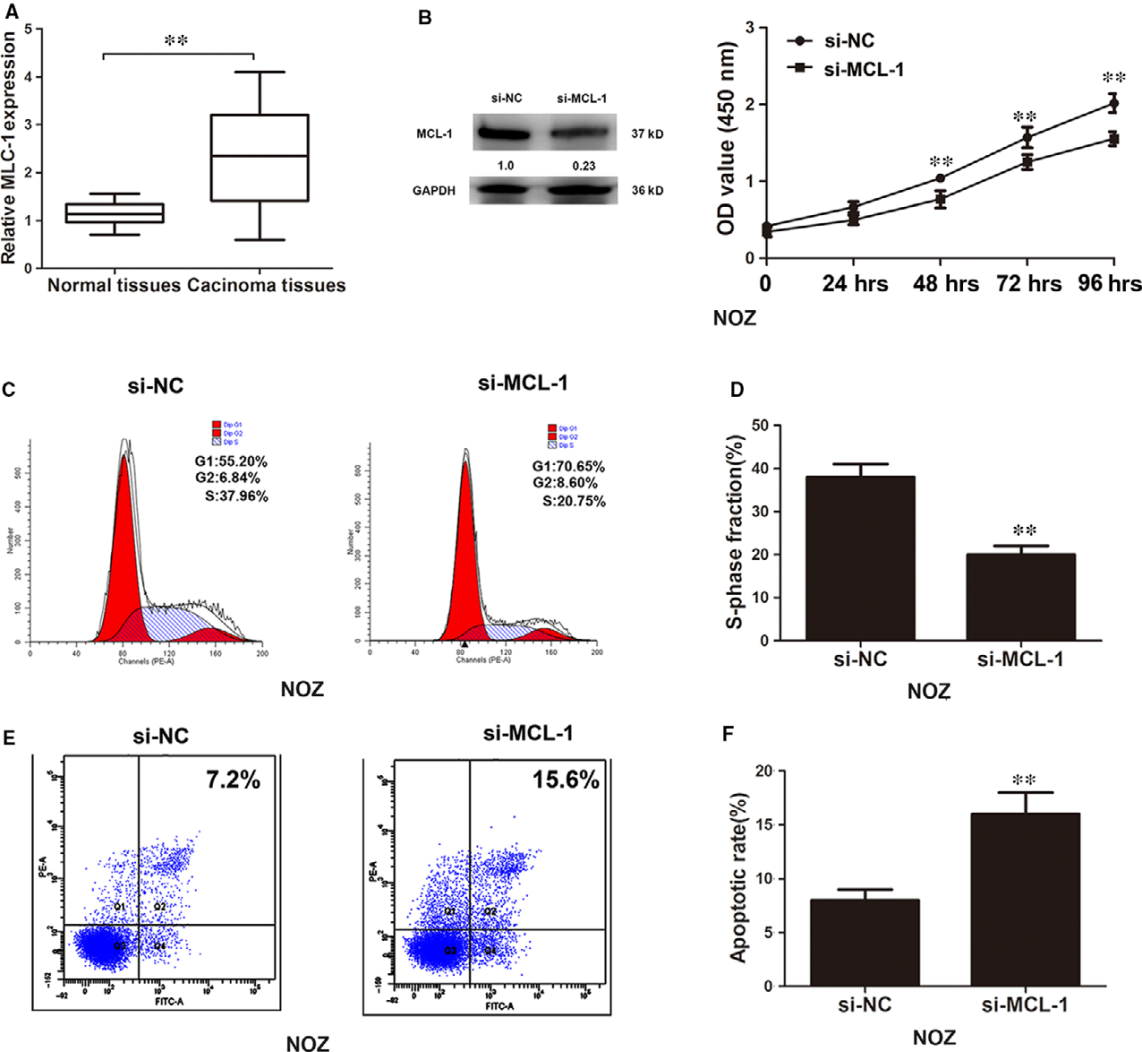
Knockdown of MCL‐1 inhibited cell proliferation, reduced S phase cell number and induced cell apoptosis. (**A**) The mRNA expression of MCL‐1 in 33 gallbladder cancer samples and adjacent normal tissues. (**B**) Cell proliferation was evaluated in NOZ cells transfected with si‐NC and si‐MCL‐1 by CCK‐8 assays after transfection at 24, 48, 72 and 96 hrs, and the absorbance at 450 nm is shown. (**C** and **D**) Cell cycle analysis of NOZ cells transfected with si‐NC and si‐MCL‐1. (**E** and **F**) Apoptosis rates were evaluated in NOZ cells transfected with si‐NC and si‐MCL‐1. Error bars represent the mean ± S.D. of triplicate experiments, **P < 0.05.

To explore the role of MCL‐1 in gallbladder cancer progression, we next measured the expression levels of MCL‐1 by qRT‐PCR. We found that MCL‐1 was up‐regulated in the tumour specimens (Fig. 6A). SiRNA‐MCL‐1 was stably introduced into NOZ cells and effectively decreased the endogenous level of MCL‐1. The growth curves detected by CCK‐8 assays showed that MCL‐1 silencing significantly decreased cell proliferation in NOZ cells (Fig. 6B). Cell cycle assays confirmed that the S phase cell numbers were significantly reduced in the MCL‐1 silencing group compared with the control group in NOZ cells (Fig. 6C and D). Annexin V‐FITC analysis showed that the proportion of the cell population undergoing apoptosis was increased after knockdown of MCl‐1 in NOZ cells (Fig. 6E and F).

### Knockdown of MALAT1 decreases tumour volumes and down‐regulates the MCL‐1 level *in vivo*

**Figure 7:**
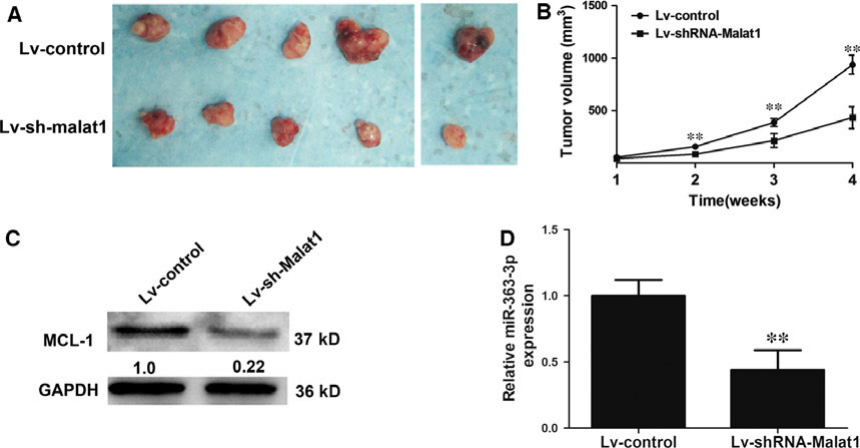
Knockdown of MALAT1 inhibited tumour growth *in vivo*. (**A**) Representative photo of tumour formation: Lv‐shRNA‐MALAT1 and Lv‐control cells were subcutaneously incubated in nude mice up to 4 weeks. (**B**) Statistical analysis of tumour volumes changes *in vivo*. (**C**) Western blot detection of MCL‐1 expression after MALAT1 silencing *in vivo*, **P < 0.05. (**D**) qRT‐PCR analysis of miR‐363‐3p expression in tumour tissues after MALAT1 silencing *in vivo*. MiR‐363‐3p was normalized to U6, and the statistical differences between samples were analysed with paired samples *t*‐test, **P < 0.05.

To further verify the above findings, we constructed NOZ cell lines stably expressing shRNA‐MALAT1 or the negative control. Then, we subcutaneously injected nude mice with NOZ cells stably silenced for MALAT1 or control cells. Tumours formed by MALAT1‐silenced cells grew much slower than those formed by control cells, and the tumour volumes from the MALAT1 knockdown group were significantly smaller than those from the control group (Fig. 7A and B). The protein level of MCL‐1 decreased in the MALAT1‐silenced group compared with that of the control group (Fig. 7C). Additionally, we found that the miR‐363‐3p level in tumour tissues was decreased (Fig. 7D). These results confirmed that MALAT1 is essential for regulating gallbladder cancer cell growth and it up‐regulated MCL‐1 expression by sponging miR‐363‐3p *in vivo*.

## Discussion

Metastasis‐associated lung adenocarcinoma transcript 1 is an important long non‐coding RNA in tumour progression. It was previously reported that silencing of MALAT1 in human gallbladder cancer cells suppressed cell proliferation and invasion 13. In this study, qRT‐PCR analysis showed that MALAT1 expression levels were significantly increased in gallbladder cancer samples compared with adjacent normal tissues. Knockdown of MALAT1 inhibited gallbladder cancer cell proliferation, induced cell apoptosis and decreased the tumour volume *in vivo*. These findings indicated that MALAT1 may function as an oncogene, and its overexpression could contribute to gallbladder cancer development.

Recently, a growing number of reports have suggested that lncRNAs act as ‘sponges’ to bind specific miRNAs and regulate their function. Yang and his team demonstrated that depletion of UCA1 was involved in the down‐regulation of matrix metallopeptidase 14 (MMP14), a target gene of miR‐485‐5p. These results suggested that UCA1 is a new prognostic biomarker for epithelial ovarian cancer (EOC) and established a novel connection among UCA1, miR‐485‐5p and MMP14 in EOC metastasis 17. In pancreatic adenocarcinoma, the lncRNA ROR was shown to act as a ceRNA, and decreased ROR expression could inhibit cell proliferation, invasion and tumorigenicity by modulating Nanog and sponging miR‐145 18. Recently, a study demonstrated that a novel lncRNA, C032469, which was highly expressed in gastric cancer tissues, could directly bind to miR‐1207‐5p, effectively functioned as a sponge for miR‐1207‐5p to modulate the expression of hTERT 19. Similarly, the lncRNA MALAT1 was reported to function as a competing endogenous RNA to regulate ZEB2 expression by sponging miR‐200s in clear cell kidney carcinoma 9.

However, the ceRNA mechanisms for MALAT1 deregulation in gallbladder cancer have not been thoroughly elucidated. Here, we showed that MALAT1 is a target of miRNA‐363‐3p by bioinformatics analysis and luciferase reporter assays. We next demonstrated that MALAT1 was in the same RISC by RIP assays. Furthermore, we used RNA pull‐down assays to explore whether MALAT1 can be pulled down by biotinylated miR‐363‐3p. The results indicated that introduction of mutated biotinylated miR‐363‐3p disrupted base pairing between MALAT1 and miR‐363‐3p, which led to the inability of miR‐363‐3p to pull down MALAT1, but biotinylated miR‐363‐3p could pull down the MALAT1. These experiments demonstrated that MALAT1 is a ceRNA sponge of miR‐363‐3p. QRT‐PCR and Western blot analyses also showed that knockdown of MALAT1 resulted in a significant decrease in endogenous MCL‐1 mRNA and protein expression by binding to miR‐363‐3p in SGC‐996 and NOZ cells. Moreover, knockdown of MALAT1 inhibited cell proliferation, decreased the S phase cell population and increased cell apoptosis by sponging miR‐363‐3p.

Myeloid cell leukaemia‐1 is a highly expressed anti‐apoptotic Bcl‐2 protein in cancer that protects cells from apoptosis by binding to Bax and Bak, pro‐apoptotic members of the Bcl‐2 family, thereby blocking mitochondrial outer membrane permeabilization, cytochrome c release, and the activation of the caspase cascade 20. Increased expression of MCL‐1 has been shown to contribute to carcinogenesis, inhibit apoptosis and cell cycle progression and promote cancer cell replication, invasion, metastasis and chemoresistance 21, 22, 23, 24. Our results demonstrated that inhibition of MCL‐1 suppressed cell proliferation, decreased the number of cells in S phase and increased cell apoptosis. Therefore, the effect of MALAT1 on gallbladder cancer cell proliferation is due, in part, to its function as a molecular sponge of miR‐363‐3p that targets MCL‐1.

In summary, MALAT1 functioned as a miRNA sponge to attenuate the endogenous function of miR‐363‐3p, which negatively modulates MCL‐1 expression. Targeting the ceRNA regulatory network may be a novel therapeutic strategy for gallbladder cancer. A future study using a large cohort of samples from gallbladder cancer patients is needed to confirm the prognostic value of MALAT1 in GBC patients.

## Conflict of interest

The authors declare no conflict of interest.

## References

[1] Gourgiotis S , Kocher HM , Solaini L , *et al* Gallbladder cancer. Am J Surg. 2008; 196: 252–64. 18466866 10.1016/j.amjsurg.2007.11.011

[2] Hawkins WG , DeMatteo RP , Jarnagin WR , *et al* Jaundice predicts advanced disease and early mortality in patients with gallbladder cancer. Ann Surg Oncol. 2004; 11: 310–5. 14993027 10.1245/aso.2004.03.011

[3] Huarte M . The emerging role of lncRNAs in cancer. Nat Med. 2015; 21: 1253–61. 26540387 10.1038/nm.3981

[4] Zhang H , Chen Z , Wang X , *et al* Long non‐coding RNA: a new player in cancer. J Hematol Oncol. 2013; 6: 37. 23725405 10.1186/1756-8722-6-37 PMC3693878

[5] Ji P , Diederichs S , Wang W , *et al* MALAT‐1, a novel noncoding RNA, and thymosin beta4 predict metastasis and survival in early‐stage non‐small cell lung cancer. Oncogene. 2003; 22: 8031–41. 12970751 10.1038/sj.onc.1206928

[6] Lai MC , Yang Z , Zhou L , *et al* Long non‐coding RNA MALAT‐1 overexpression predicts tumor recurrence of hepatocellular carcinoma after liver transplantation. Med Oncol. 2012; 29: 1810–6. 21678027 10.1007/s12032-011-0004-z

[7] Okugawa Y , Toiyama Y , Hur K , *et al* Metastasis‐associated long non‐coding RNA drives gastric cancer development and promotes peritoneal metastasis. Carcinogenesis. 2014; 35: 2731–9. 25280565 10.1093/carcin/bgu200 PMC4247518

[8] Yang L , Bai HS , Deng Y , *et al* High MALAT1 expression predicts a poor prognosis of cervical cancer and promotes cancer cell growth and invasion. Eur Rev Med Pharmacol Sci. 2015; 19: 3187–93. 26400521

[9] Xiao H , Tang K , Liu P , *et al* LncRNA MALAT1 functions as a competing endogenous RNA to regulate ZEB2 expression by sponging miR‐200s in clear cell kidney carcinoma. Oncotarget. 2015; 6: 38005–15. 26461224 10.18632/oncotarget.5357 PMC4741980

[10] Cao X , Zhao R , Chen Q , *et al* MALAT1 might be a predictive marker of poor prognosis in patients who underwent radical resection of middle thoracic esophageal squamous cell carcinoma. Cancer Biomark. 2015; 15: 717–23. 26406400 10.3233/CBM-150513 PMC12965489

[11] Qi Y , Ooi HS , Wu J , *et al* MALAT1 long ncRNA promotes gastric cancer metastasis by suppressing PCDH10. Oncotarget. 2016; 7: 12693–703. 26871474 10.18632/oncotarget.7281 PMC4914315

[12] Hu ZY , Wang XY , Guo WB , *et al* Long non‐coding RNA MALAT1 increases AKAP‐9 expression by promoting SRPK1‐catalyzed SRSF1 phosphorylation in colorectal cancer cells. Oncotarget. 2016; 7: 11733–43. 26887056 10.18632/oncotarget.7367 PMC4905507

[13] Wu XS , Wang XA , Wu WG , *et al* MALAT1 promotes the proliferation and metastasis of gallbladder cancer cells by activating the ERK/MAPK pathway. Cancer Biol Ther. 2014; 15: 806–14. 24658096 10.4161/cbt.28584 PMC4049796

[14] Wang K , Long B , Zhou LY , *et al* CARL lncRNA inhibits anoxia‐induced mitochondrial fission and apoptosis in cardiomyocytes by impairing miR‐539‐dependent PHB2 downregulation. Nat Commun. 2014; 5: 3596. 24710105 10.1038/ncomms4596

[15] Qu J , Li M , Zhong W , *et al* Competing endogenous RNA in cancer: a new pattern of gene expression regulation. Int J Clin Exp Med. 2015; 8: 17110–6. 26770304 PMC4694204

[16] Ou Y , Zhai D , Wu N , *et al* Downregulation of miR‐363 increases drug resistance in cisplatin‐treated HepG2 by dysregulating Mcl‐1. Gene. 2015; 572: 116–22. 26143754 10.1016/j.gene.2015.07.002

[17] Yang Y , Jiang Y , Wan Y , *et al* UCA1 functions as a competing endogenous RNA to suppress epithelial ovarian cancer metastasis. Tumour Biol. 2016; Doi:10.1007/s13277‐016‐4917‐1 10.1007/s13277-016-4917-1 26867765

[18] Gao S , Wang P , Hua Y , *et al* ROR functions as a ceRNA to regulate Nanog expression by sponging miR‐145 and predicts poor prognosis in pancreatic cancer. Oncotarget. 2016; 7: 1608–18. 26636540 10.18632/oncotarget.6450 PMC4811484

[19] Lu MH , Tang B , Zeng S , *et al* Long noncoding RNA BC032469, a novel competing endogenous RNA, upregulates hTERT expression by sponging miR‐1207‐5p and promotes proliferation in gastric cancer. Oncogene. 2015; Doi: 10.1038/onc.2015.413 10.1038/onc.2015.413 26549025

[20] Chipuk JE , Moldoveanu T , Llambi F , *et al* The BCL‐2 family reunion. Mol Cell. 2010; 37: 299–310. 20159550 10.1016/j.molcel.2010.01.025 PMC3222298

[21] Lee WS , Park YL , Kim N , *et al* Myeloid cell leukemia‐1 is associated with tumor progression by inhibiting apoptosis and enhancing angiogenesis in colorectal cancer. Am J Cancer Res. 2015; 5: 101–13. 25628923 PMC4300705

[22] Thomas LW , Lam C , Edwards SW . Mcl‐1; the molecular regulation of protein function. FEBS Lett. 2010; 584: 2981–9. 20540941 10.1016/j.febslet.2010.05.061

[23] Akgul C . Mcl‐1 is a potential therapeutic target in multiple types of cancer. Cell Mol Life Sci. 2009; 66: 1326–36. 19099185 10.1007/s00018-008-8637-6 PMC11131550

[24] Mandelin AM 2nd , Pope RM . Myeloid cell leukemia‐1 as a therapeutic target. Expert Opin Ther Targets. 2007; 11: 363–73. 17298294 10.1517/14728222.11.3.363

